# Numerical simulations of patient-specific models with multiple plaques in human peripheral artery: a fluid-structure interaction analysis

**DOI:** 10.1007/s10237-020-01381-w

**Published:** 2020-09-11

**Authors:** Danyang Wang, Ferdinand Serracino-Inglott, Jiling Feng

**Affiliations:** 1 Department of Engineering, Manchester Metropolitan University, Manchester, UK; 2 Manchester Royal Infirmary, Manchester University NHS Foundation Trust, Manchester, UK

**Keywords:** Femoral artery, Hyperelastic, Fluid-structure interaction, Patient-specific, Computed tomography

## Abstract

Atherosclerotic plaque in the femoral is the leading cause of peripheral artery disease (PAD), the worse consequence of which may lead to ulceration and gangrene of the feet. Numerical studies on fluid-structure interactions (FSI) of atherosclerotic femoral arteries enable quantitative analysis of biomechanical features in arteries. This study aims to investigate the hemodynamic performance and its interaction with femoral arterial wall based on the patient-specific model with multiple plaques (calcified and lipid plaques). Three types of models, calcification-only, lipid-only and calcification-lipid models, are established. Hyperelastic material coefficients of the human femoral arteries obtained from experimental studies are employed for all simulations. Oscillation of WSS is observed in the healthy downstream region in the lipid-only model. The pressure around the plaques in the two-plaque model is lower than that in the corresponding one-plaque models due to the reduction of blood flow domain, which consequently diminishes the loading forces on both plaques. Therefore, we found that stress acting on the plaques in the two-plaque model is lower than that in the corresponding one-plaque models. This finding implies that the lipid plaque, accompanied by the calcified plaque around, might reduce its risk of rupture due to the reduced the stress acting on it.

## Introduction

Peripheral artery disease (PAD) strikes 202 million men and women worldwide, and its prevalence has a significant growth with the increase in age (Benjamin et al. 2019). Atherosclerotic plaques build-up inside the arterial wall that diminishes the blood supply to lower extremities is the leading cause of lower limb PAD. Two significant clinical symptoms of lower limb PAD are claudication and critical limb ischemia, which may even lead to ulceration and gangrene of the toes or feet (Pande and Creager 2018). Besides, patients diagnosed with PAD have an increased risk of cardiovascular morbidity and mortality compared with those without PAD (McDermott 2015).

Several experimental studies have examined the composition and mechanical properties of femoral plaques. The experimental study by Herisson et al. (2011) showed that the components of femoral plaques and carotid plaques are different, and femoral plaques demonstrate higher calcium and lesser cholesterol concentrations than carotid plaques. Cunnane et al. (2015a) carried uniaxial mechanical testing on twenty human femoral plaques, which were classified as heavily, moderately and lightly calcified plaques based on Fourier transform infrared (FTIR) spectroscopy analysis.

Computational studies analyse the mechanism of atherosclerotic plaques, indicate that changes in the geometry and material property of a model could affect its mechanical behaviour (Holzapfel et al. 2014; Cunnane et al. 2015b; Karimi et al. 2014a). Computer fluid dynamics (CFD) modelling which enables quantitative analysis of hemodynamic factors demonstrated that low and oscillated wall shear stress (WSS) promotes the formation of atherosclerotic plaques (Ku et al. 1985; Olgac et al. 2009; Timmins et al. 2017), whereas high WSS can increase the vulnerability of plaques (Samady et al. 2011). Finite element (FE) simulations allow analysing plaque stresses to investigate plaque vulnerability (Cardoso et al. 2014; Gao et al. 2009; Kock et al. 2008). Rupture of vulnerable plaques may happen when the external loading excesses the plaques strength (Rogers et al. 2000; Fernández-Ortiz et al. 1994). von Mises stress (VMS) is commonly accepted as a useful measurement in evaluating the maximal stress in plaques (Holzapfel et al. 2014; Humphrey and Holzapfel 2012).

The anatomic geometries of atherosclerotic plaques influence the hemodynamic features of the blood flow, which in turn affects the mechanical behaviour of the plaques. To this end, fluid-structure interaction (FSI) simulation, which couples computational fluid dynamics with structure mechanics, provides a useful way in biomechanical analysis. Recent studies of 3D FSI simulations have been implemented for coronary, carotid and femoral atherosclerotic artery models (Tang et al. 2004, 2005; Karimi et al. 2014a; Kim et al. 2008). Karimi et al. (2014a) conducted an FSI simulation for a coronary artery with three types of plaques. They concluded that different plaque types affect plaque stress prominently. The lowest VMS was observed on the calcified plaque, while the highest VMS was observed on the hypocellular plaque. Kim et al. (2008) carried a 3D FSI analysis with a patient-specific femoral artery bifurcation model. Both FSI and CFD simulations were conducted to investigate the influence of a compliant wall on the fluid dynamics. Their results indicate that there is no significant influence of the wall motion on the global fluid dynamics characteristics.

To the best of author’s knowledge, FSI simulations concerning multiple plaques in a realistic model were not reported for human femoral arteries. In this paper, we aim to discuss biomechanical characteristics for an atherosclerotic femoral artery in three models: blood coupling with artery wall and calcified plaque; blood coupling with artery wall and lipid plaque; and blood coupling with artery wall, calcified and lipid plaques. Appropriate hyperelastic material parameters obtained from experimental studies are adopted to the femoral arterial wall and plaques. A one-way FSI coupling is employed for patient-specific stenosed femoral artery models, which are generated based on computed tomography (CT) data to provide more realistic artery and plaque geometries for the simulations. Pulsatile boundary conditions are used in this study.

## Materials and methods

### Geometry reconstruction

**Fig. 1:**
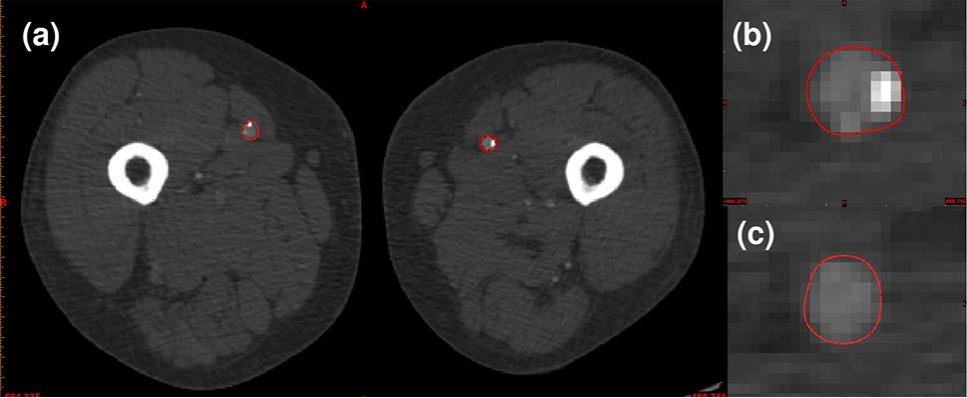
CT scans of the patient’s lower limb in axial view. (**a**) A CT scan of the patient’s lower limb. (**b**) A profile of the left femoral artery lumen with calcified plaque, marked by a red line. (**c**) A profile of the left femoral artery lumen without plaque, marked by a red line

The CT images of a 73-year-old male patient with stenosed lower limb vessels are used to construct a patient-specific 3D femoral artery model. The data contain 3918 slices of CT scans with 0.625 mm slice thickness. Each slice has $512\times 512$ pixels. Figure 1a shows a CT slice of the patient’s lower limb, the two large bright domains are femora, and the domain marked by two red lines is the lumen area in the femoral artery. The calcified plaque, which is brighter than the lumen area, can be identified from the CT scan (shown in Fig. 1a, b). Figure 1c shows a healthy lumen profile in the left femoral artery (marked by the red line). Notably, the artery wall cannot be segmented from CT scans directly. The CT images were identified and selected by our clinical collaborator. Ethical approval was obtained from HRA and Health and Care Research Wales (HCRW).

**Fig. 2:**
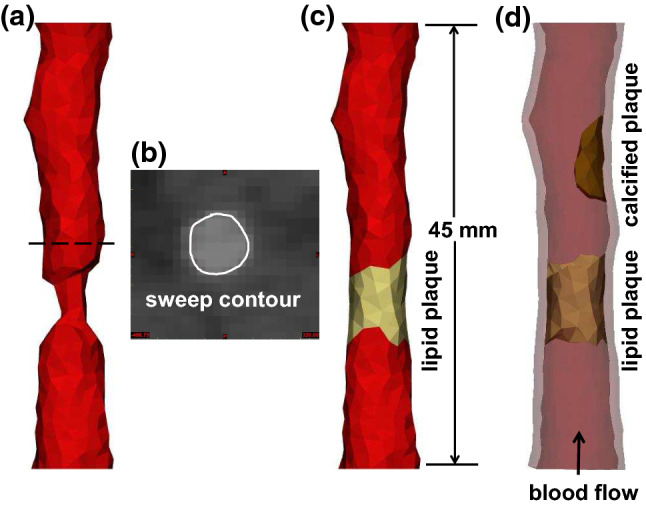
Three-dimensional structure-fluid geometry of left femoral artery. (**a**) The lumen geometry, the dashed line represents the location of a sweep contour shown in Fig. 2b. (**b**) The sweeping contour of a healthy lumen area (solid white line). (**c**) The 3D geometry of lipid plaque and lumen. (**d**) The 3D geometry of the artery wall, calcified and lipid plaques

A 45-mm-long left femoral artery model is reconstructed based on these CT scans, two types of plaque are identified, shown in Fig. 2d. Materialise Mimics 22.0 is used to form the 3D models. First, we build the lumen geometry from the CT scans and discover a significant narrowing in a near the upstream of the lumen (shown in Fig. 2a). The CT scans of the narrowing area show no existence of calcified plaque. Hence, we assume that this is the place where lipid plaque develops. In order to construct the lipid plaque geometry, a healthy lumen contour (Fig. 2b) from the CT slice that is located in the dashed line in Fig. 2a is selected as the sweep contour. Then, an estimated lumen geometry at the stenotic lesion is built by sweeping the contour along the lumen centreline. Following this, we construct the lipid plaque using this estimated lumen geometry eliminating the original lumen narrowing domain, shown in Fig. 2c. Finally, an artery wall of 1 mm thickness is designed based on the surfaces of the lumen and the lipid plaque (Fig. 2d). The arterial wall thickness is adopted based on the previously reported study (Persson et al. 1992). In addition, a calcified plaque sits near the downstream of the artery is identified from the CT scans (Fig. 2d). The stenosis degree (based on diameter) of the lipid plaque (about $61\%$) is higher than that of the calcified plaque (about $43\%$).

Using the geometry built above, we construct three models: the calcification-only model, which is consist of the calcified plaque, the arterial wall and the corresponding blood domain; the lipid-only model, which comprises the lipid plaque, the arterial wall and the corresponding blood domain; the calcification-lipid model, which contains the calcified, lipid plaques, the arterial wall and the corresponding blood domain.

### Material property

The femoral artery wall is assumed to be nonlinear, homogenous and hyperelastic material. The strain energy function *W* of Mooney–Rivlin constitutive model is employed:

1 $$\begin{aligned} W&=a_{10}\left( I_1-3\right) +a_{01}\left( I_2-3\right) +{a_{20}\left( I_1-3\right) ^2},\nonumber \\&+a_{11}\left( I_1-3\right) \left( I_2-3\right) +a_{02}\left( I_2-3\right) ^2 \end{aligned}$$

where $I_1$ and $I_2$ are first and second strain invariants. The coefficients $a_{10}$, $a_{01}$, $a_{20}$, $a_{11}$ and $a_{02}$ are determined based on experimental data of human femoral artery (Prendergast et al. 2003). The strain energy function of Yeoh hyperelastic material model is used for the lipid and calcified plaques as:

2 $$\begin{aligned} W=c_{10}\left( I_1-3\right) +c_{20}\left( I_1-3\right) ^2+c_{30}\left( I_1-3\right) ^3. \end{aligned}$$

The value of material constants $c_{10}$, $c_{20}$ and $c_{30}$ are chosen based on the experimental data of human atherosclerotic plaque for stenosed femoral artery (Cunnane et al. 2015a, b). Cunnane et al. (2015a, (2015b) grouped their tested plaque samples as ‘lightly calcified,’ ‘moderately calcified’ and ‘heavily calcified’ plaques using Ca:Li ratio, which biologically characterises the calcified and lipid contents within plaques. Based on the patient’s CT scans, there is no significant calcified tissue in the lipid plaques, whereas the calcification in the calcified plaque is notable. Hence, we use the material coefficients of ‘lightly calcified’ and ‘heavily calcified’ plaques from Cunnane et al. (2015b) as the lipid and calcified plaques material parameters in this study, respectively.

**Fig. 3:**
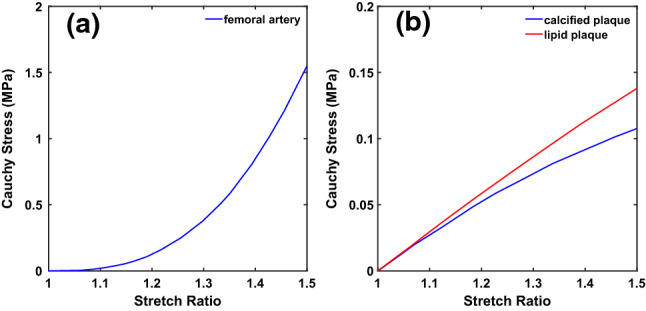
Reconstructed uniaxial Cauchy stress–stretch ratio behaviour of femoral artery and plaques (Prendergast et al. 2003; Cunnane et al. 2015a, b). (**a**) The Cauchy stress–stretch ratio behaviour of femoral artery; (**b**) The Cauchy stress–stretch ratio behaviour of calcified and lipid plaques

Blood is considered as non-Newtonian fluid with constant density $1050~{\mathrm{kg/m}}^3$. The Carreau model is employed for the blood (Cho and Kensey 1991):

3 $$\begin{aligned} \mu =\mu _{\infty }+\left( \mu _0-\mu _{\infty }\right) \left( \lambda \dot{\gamma }\right) ^{\frac{n-2}{2}}, \end{aligned}$$

where $\mu$, $\mu _0$ and $\mu _{\infty }$ are the blood viscosity, blood viscosity at zero shear rate and blood viscosity at infinite shear rate, respectively. The material coefficient $\lambda$ and ${\dot{\gamma }}$ represent the relaxation time and shear rate, and *n* is a power index. The values of the material parameters used in the simulations are listed in Table 1. The reconstructed Cauchy stress–stretch ratio curves from uniaxial mechanical testing for the femoral artery, calcified and lipid plaques are shown in Fig. 3.

**Table 1:** Material parameters of blood, femoral artery, femoral lipid and calcified plaque

Material	Model	Material parameters
Blood (Cho and Kensey (1991))	Carreau	$\lambda =3.313$ s, $n=0.3568$, $\mu _0=0.056~{\mathrm{Pa}}\cdot {\mathrm{s}}$, $\mu _{\infty }=0.00345~{\mathrm{Pa}}\cdot {\mathrm{s}}$
Artery (Prendergast et al. (2003))	Mooney–Rivlin	$a_{10}=18.9~{\mathrm{kPa}}$, $a_{01}=2.75~{\mathrm{kPa}}$, $a_{20}=590.42~{\mathrm{kPa}}$, $a_{11}=857.18~{\mathrm{kPa}}$,$a_{02}=0~{\mathrm{kPa}}$
Lipid plaque (lightly calcified (Cunnane et al. 2015b))	Yeoh	$c_{10}=49.8~{\mathrm{kPa}}$, $c_{20}=-6.19~\mathrm{kPa}$, $c_{30}=0.898~\mathrm{kPa}$
Calcified plaque (heavily calcified (Cunnane et al. 2015b))	Yeoh	$c_{10}=46.2~\mathrm{kPa}$, $c_{20}=-14.7~\mathrm{kPa}$, $c_{30}=4.95~\mathrm{kPa}$

The parameters of lipid and calcified plaques are based on the parameters of ‘lightly calcified’ and ‘heavily calcified’ femoral plaques from the literature Cunnane et al. (2015b)

### Fluid-structure interaction and boundary conditions

**Fig. 4:**
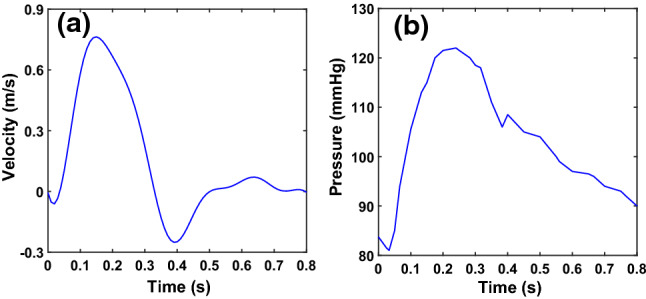
Reconstructed time-dependent boundary conditions waveform (Olufsen et al. 2000). (**a**) Inlet velocity waveform and (**b**) outlet pressure waveform

One-way FSI simulations are performed, which pass the pressure obtained from the CFD simulation to the interaction surface (i.e. the surface of the lumen domain) for mechanical simulation at each time step. All simulations are performed in ANSYS® Workbench 19.2, ANSYS® Fluent is used for CFD simulations, and structure analysis is performed in ANSYS® Academic Research Mechanical. We assume ‘bound contacts’ between the artery wall and plaques, i.e. no separation and penetration between these structures. The two ends of the artery wall are fixed in all directions. A uniformed pressure 1 kPa is applied on the outer surface of the artery wall to diminish possible oscillations, which may cause divergence of simulations. Time-dependent inlet velocity and outlet pressure boundary conditions are used for all simulations (Fig. 4 ). The pulsatile velocity and pressure waveforms of blood flow in human femoral artery are obtained from the literature (Olufsen et al. 2000). Simulation time equals to one cardiac cycle 0.8 s, and the peak velocity and pressure appear at time 0.16 s and 0.23 s, respectively. The models are discretized in ANSYS® MESH with patch independent tetrahedron elements. For calcification-lipid model, the fluid domain contains 295742 elements and the solid domain (arterial wall and plaques) contains 160882 elements. Same mesh method and size are used for calcification-only and lipid-only models. For all three models, the mesh skewness is about 0.6.

## Results

To investigate how plaques with different anatomic geometry and material property affect the biomechanical characteristics of the models, we demonstrate the results in three models: blood coupling with artery and calcified plaque (calcification-only model); blood coupling with artery and lipid plaque (lipid-only model); and blood coupling with artery, calcified and lipid plaques (calcification-lipid model).

### Blood flow pattern

**Fig. 5:**
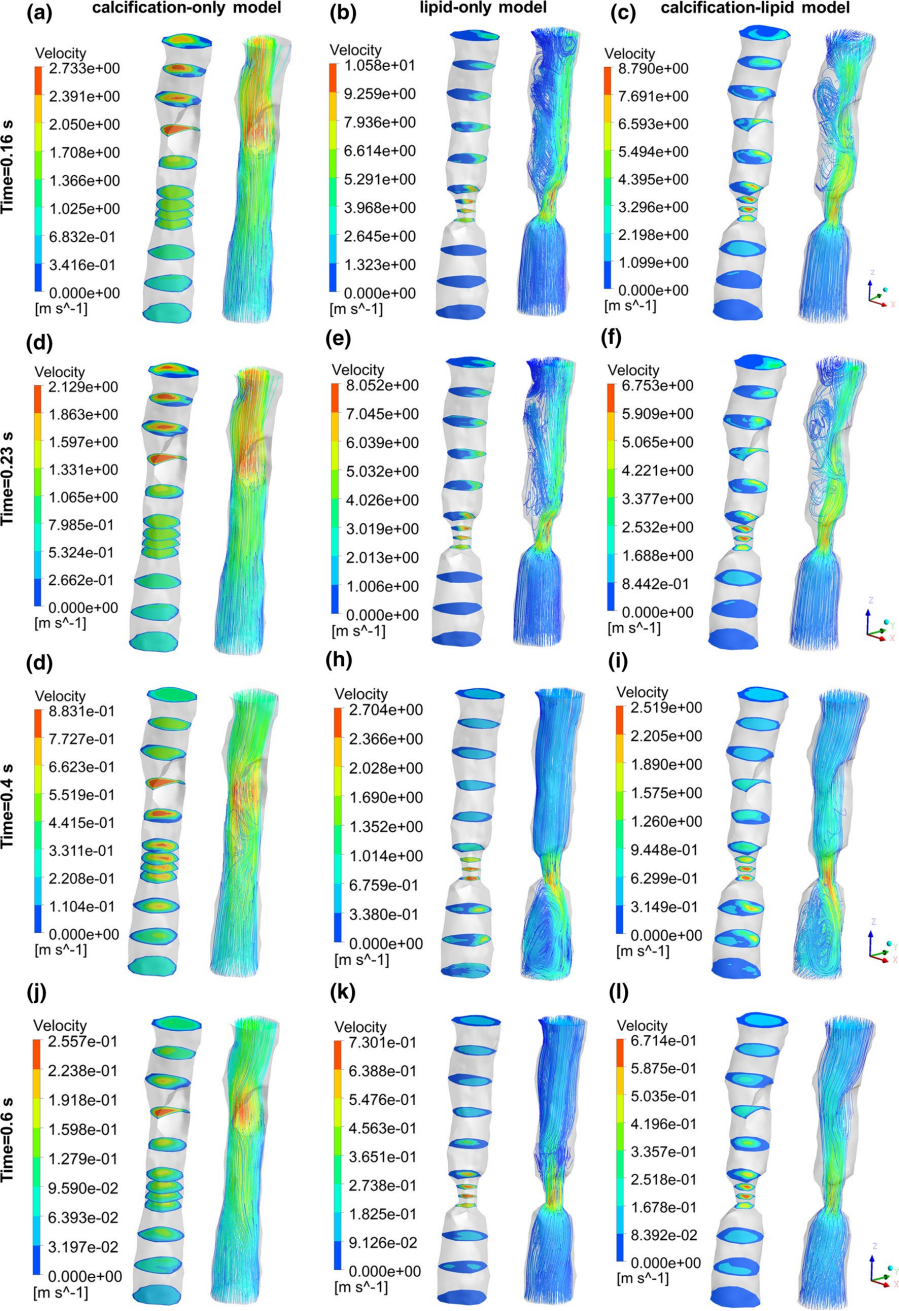
Velocity contours and streamlines in three models at various time. (**a**), (**d**), (**g**), (**i**) Velocity contours and streamlines of calcification-only model at time 0.16 s, 0.23 s, 0.4 s and 0.6 s; (**b**), (**e**), (**h**), (**k**) velocity contours and streamlines of lipid-only model at time 0.16 s, 0.23 s, 0.4 s and 0.6 s; (**c**), (**f**), (**i**), (**l**) velocity contours and streamlines of calcification-lipid model at time 0.16 s, 0.23 s, 0.4 s and 0.6 s

Figure 5 displays the flow velocity contours and the corresponding streamline at time 0.16 s, 0.23 s (peak systole), 0.4 s and 0.6 s in calcification-only model (Fig. 5a, d, g, j), lipid-only model (Fig. 5b, e, h, k) and calcification-lipid model (Fig. 5c, f, i, l). The maximal velocity appears in the stenosis region in all models as expected. Additionally, the maximal velocity in calcification-only model is significantly lower than the maximal velocity in lipid-only model and calcification-lipid model at all times. The flow in lipid-only model and calcification-lipid model indicates a similar pattern. However, the maximal velocity in lipid-only model is higher than that in calcification-lipid model at all times. Several complex vortices appear in the flow with plaque (lipid-only model and calcification-lipid model), and these vortices mainly located in the downstream of the lipid plaque (Fig. 5b, e and c, f). The blood is generally flowed from the inlet to the outlet during one cardiac cycle. However, the flow profiles of the models with lipid plaque show recirculation around the lipid plaque, especially at 0.4 s (Fig. 5h, i).

### Wall shear stress and time-averaged wall shear stress

**Fig. 6:**
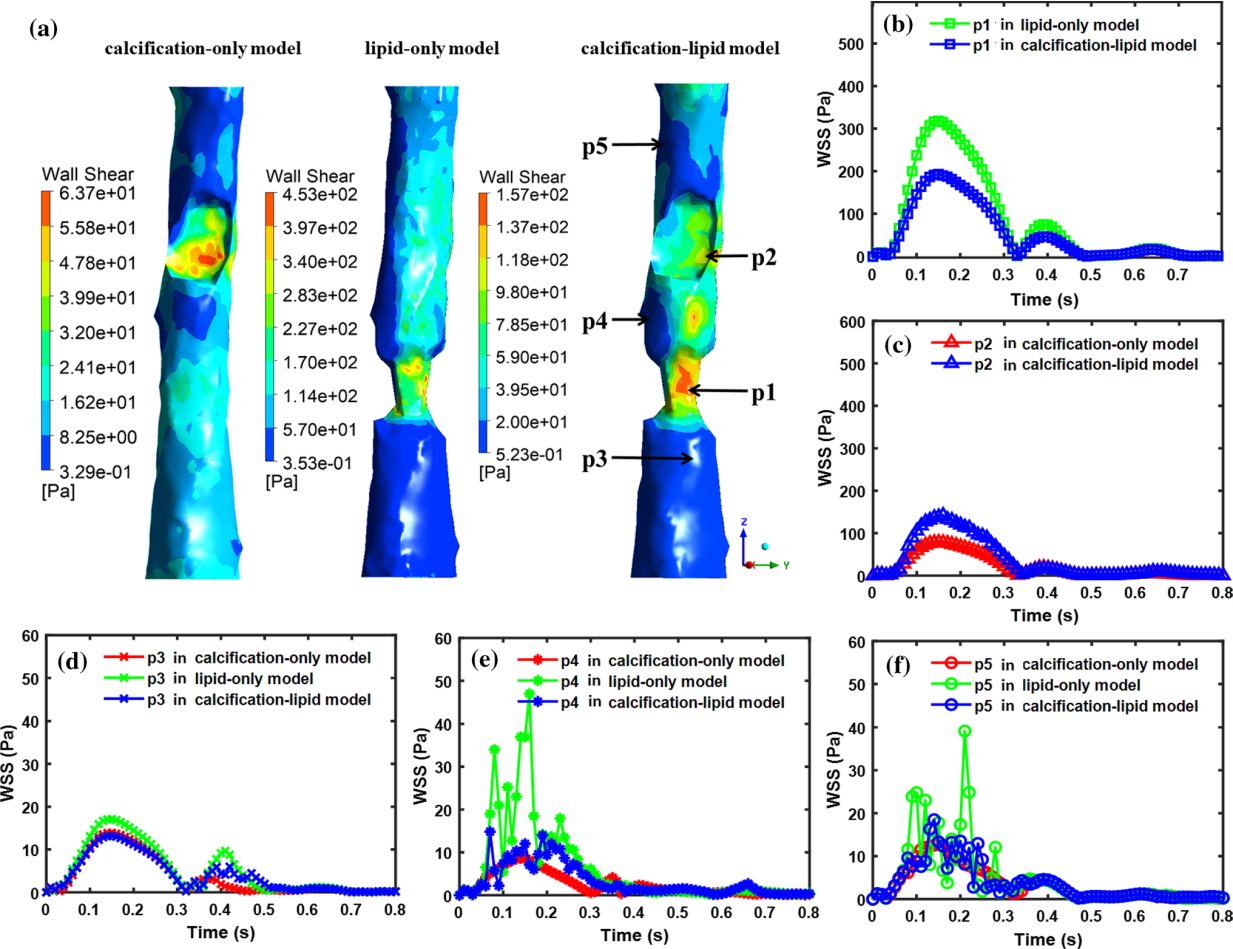
WSS results in three models. (**a**) WSS distribution in calcification-only model, lipid-only model and calcification-lipid model at peak systole, respectively; (**b**)–(**f**) Time evolutions of WSS on p1–p5 (shown in Fig. 4a) in calcification-only model, lipid-only model and calcification-lipid model

Figure 6 shows the WSS results for the blood domain. The WSS distributions at peak systole in three models are in Fig. 6a. The WSS concentrates in the stenosis region in three models, which coincides with the observations from the literatures (Cilla et al. 2015; Gao et al. 2009). Time evolutions of WSS in one cardiac cycle at five chosen points (p1–p5 shown in Fig. 6a) in three models are in Fig. 6b–f. Points p1 and p2 are located in the lipid and calcified plaque surfaces, respectively. And points p3, p4 and p5 are located in the healthy lumen area. Red, green and blue lines represent the time evolutions of WSS in calcification-only model, lipid-only model and calcification-lipid model, respectively. The WSS evolutions in Fig. 6b–f follow a similar trend except for that at p4 and p5 in lipid-only model and calcification-lipid model, which oscillate rapidly during systole. Figure 6b indicates that the presence of the calcified plaque (calcification-lipid model) can diminish the WSS value around the lipid plaque, whereas the presence of lipid plaque (calcification-lipid model) can increase the WSS around the calcified plaque (Fig. 6c). However, there is no significant difference in WSS magnitude on p3, p4 and p5 in three models. Time-averaged WSS (TAWSS) is measured and calculated in the form:

4 $$\begin{aligned} \mathrm{TAWSS}=\frac{1}{T}\int _0^T\left| \mathbf {\tau }_{\omega }(t)\right| \,\mathrm{d}t, \end{aligned}$$

where *T* denotes one cardiac cycle, $\mathbf {\tau _{\omega }}(t)$ and $\left| \mathbf {\tau _{\omega }}(t)\right|$ represent the instantaneous WSS vector and the WSS value at time *t*, respectively. The values of TAWSS on each point are listed in Table 2. The TAWSS on p1 and p2 are higher than that on p3, p4 and p5 in all models. Notably, the TAWSS on p1 is significantly higher than the TAWSS on p2.

**Table 2:** Values of TAWSS on p1, p2, p3, p4 and p5 in three models

Models	p1	p2	p3	p4	p5
Calcification-only model	None	$20.86~\mathrm{Pa}$	$3.65~\mathrm{Pa}$	$2.42~\mathrm{Pa}$	$3.50~\mathrm{Pa}$
Lipid-only model	$82.36~\mathrm{Pa}$	None	$5.02~\mathrm{Pa}$	$6.05~\mathrm{Pa}$	$4.64~\mathrm{Pa}$
Calcification-lipid model	$52.21~\mathrm{Pa}$	$35.31~\mathrm{Pa}$	$4.03~\mathrm{Pa}$	$3.31~\mathrm{Pa}$	$3.70~\mathrm{Pa}$

### Time evolutions of blood pressure

**Fig. 7:**
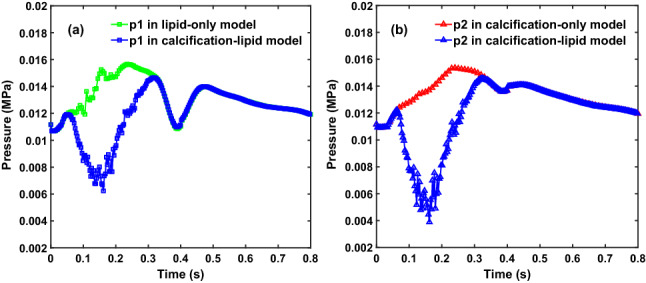
Time evolutions of blood pressure on p1 and p2 in three models. (**a**) Time evolutions of blood pressure on p1 in lipid-only model and calcification-lipid model; (**b**) time evolutions of blood pressure on p2 in calcification-only model and calcification-lipid model

Figure 7 demonstrates the time evolutions of blood pressure on p1 and p2 in one cardiac cycle. The pressure on p1 decreases dramatically in calcification-lipid model during systole and then increases until eventually equal to the pressure in lipid-only model during diastole (Fig. 7a). Similarly, the pressure on p2 in calcification-lipid model decreases prominently and then increases until it reaches the pressure in calcification-only model during diastole (Fig. 7b). Notably, the pressures on p1 and p2 in the model with two plaques (calcification-lipid model) are lower than that in the model with one plaque (lipid-only model and calcification-only model, respectively) during systole.

### von Mises stress distribution

**Fig. 8:**
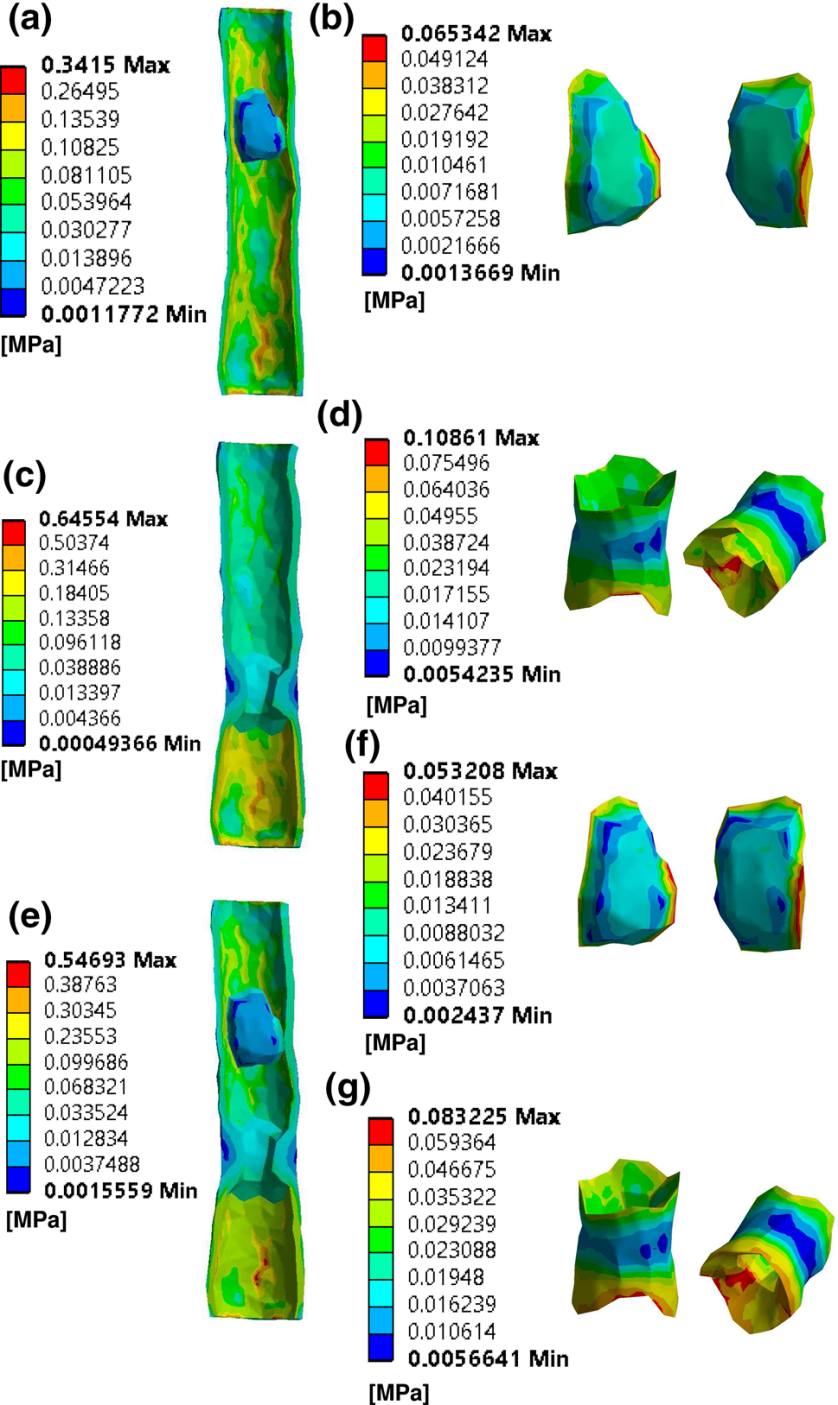
VMS distribution in three models at peak systole. (**a**), (**b**) VMS distribution of artery and calcified plaque in calcification-only model; (**c**), (**d**) VMS distribution of artery and lipid plaque in lipid-only model; (**e**), (**f**), (**g**) VMS distribution of artery, calcified plaque and lipid plaque in calcification-lipid model

Figure 8 demonstrates the VMS distributions at peak systole. The VMS distributions with the longitudinal cutting view of the models in calcification-only model, lipid-only model and calcification-lipid model are in Fig. 8a, c and e, respectively. The magnitude of VMS over the plaque is lower than that over the artery wall in general. The VMS distributions over calcified plaques exhibit a similar pattern in calcification-only model and calcification-lipid model, the maximum stresses (65.3 kPa in calcification-only model and 83.2 kPa in calcification-lipid model) located near the plaque shoulder region (Fig. 8b, f)). The plaque shoulder is commonly accepted as one of the regions that plaque disruption occurs (Pasterkamp et al. 1999; Falk et al. 1995). Figure 8d and g depicts the VMS distributions over the lipid plaque in lipid-only model and calcification-lipid model. The VMS distributions over lipid plaque also demonstrate an identical pattern. Lipid plaque stress concentrates mainly in two regions: the plaque shoulder and the region inside the upstream of the plaque. However, the maximum stresses (108.6 kPa in lipid-only model and 83.2 kPa in calcification-lipid model) sit on the inside of the lipid plaque upstream region rather than the plaque shoulder.

### Time evolutions of maximum plaque stress

**Fig. 9:**
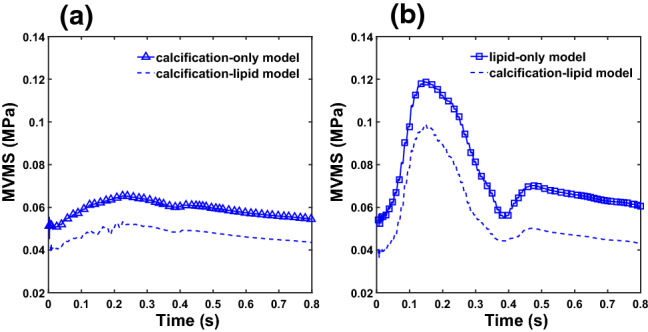
Time evolutions of MVMS over the calcified and lipid plaques in three models. (**a**) Time evolutions of MVMS over calcified plaque in calcification-only model and calcification-lipid model; (**b**) The time evolutions of MVMS over lipid plaque in lipid-only model and calcification-lipid model

We denote the (spatial) maximum VMS and the maximum principal stress over the plaques as ‘MVMS’. The time evolutions of MVMS over two plaques in all models are shown in Fig. 9. The MVMS evolutions of lipid plaque (Fig. 9b) has a notable increase during the systole compared with the MVMS evolutions of calcified plaque (Fig. 9a). The magnitude of MVMS over lipid plaque in lipid-only model (the line marked by squares in Fig. 9b) is generally higher than that over the calcified plaque in calcification-only model (the line marked by triangles in Fig. 9a). Comparable observation appears for the lipid and calcified plaques in calcification-lipid model (dashed lines in Fig. 9b and a). The highest MVMS for lipid plaque (118.7 kPa) is observed in lipid-only model, which is greater than the highest MVMS for calcified plaque (65.3 kPa) in calcification-only model. Moreover, the MVMS over the calcified plaque in calcification-lipid model is lower than that in calcification-only model (Fig. 9a) at all times. Likewise, the MVMS over the lipid plaque in calcification-lipid model is lower than that in lipid-only model (Fig. 9b) at all times.

## Discussion

Previous computational studies in biomechanical characteristics of artery models principally focused on stenotic coronary and carotid arteries. Numerical simulations based on human femoral arteries are limited. Besides, models in most of these studies considered only one plaque (Karimi et al. 2014b; Kock et al. 2008; Tang et al. 2005). So far, no extensive study investigates the biomechanical factors of a stenosed femoral artery with two types of plaques. Therefore, the primary objective of this study is to explore how plaques with different geometry and material property affect the hemodynamic and mechanical behaviours of blood and femoral plaques.

In this study, the patient-specific model of the human left femoral artery with two plaques is reconstructed based on slices from computed tomography, which is a non-invasive imaging modality widely used in clinics. This technic can identify the lumen and calcified plaques; however, it has the disadvantage of identifying lipid-rich plaques. We establish a reconstruction scheme based on CT scans, which builds not only the lumen and calcified plaque geometries but also the lipid plaque and artery wall geometries.

The CFD results (Fig. 5) indicate that complex vortices appear with the existence of lipid plaque (high stenosis degree) but not with just the calcified plaque (low stenosis degree). The maximum velocity locates in the stenosis region, as expected. Besides, the maximum velocities in calcification-lipid model (enclosed calcified and lipid plaques) are lower than that in lipid-only model (enclosed lipid plaques) but are higher than that in calcification-only model (enclosed calcified plaques). Thus, the calcified plaque can diminish the maximum velocity while the lipid plaque can raise the maximum velocity.

Maximum WSS appears in the stenosis region (Fig. 6). This observation agrees with the results in the literature (Gao et al. 2009). Similar to the observation from the maximum blood velocity in above, the presence of calcified plaque can diminish the WSS in the lipid plaque surface (Fig. 6b), whereas the presence of lipid plaque can increase the WSS in the calcified plaque surface (Fig. 6c). Conversely, the effect of calcified and lipid plaques on the WSS in the healthy region is not significant (Fig. 6d, e, f). Furthermore, the WSS oscillates rapidly at points located behind the lipid plaque (green and blue lines in Fig. 6e, f). However, there is no significant oscillation of WSS on all points in calcification-only model (red lines in Fig. 6c, d, e and f). The clinical study suggested that the blood velocity and wall shear stress oscillated in the location behind plaques in both magnitude and direction during systolic phase, which agrees with our observation Ku et al. (1985). The TAWSS on stenosis surfaces is significantly higher than that in the healthy surface. Additionally, the TAWSS on the calcified plaque surface (20.8 Pa) is lower than that on the lipid plaque surface (82.3 Pa). Hence, higher stenosis produces higher WSS, which is coincident with the observation from the literature (Gholipour et al. 2018).

Gholipour et al. (2018) performed a fluid-structure interaction simulation for a coronary artery with an idealized single-plaque model. They investigate the risk of plaque rupture with varying levels of stenosis and tapered shape of the artery. Their results displayed that both significant levels of plaque stenosis and tapered shape of the artery contribute to high wall shear stress. For models with stenosis levels of $25\%$, $35\%$, $45\%$ and $55\%$, the maximum WSS are 96 Pa, 126 Pa, 150 Pa and 172 Pa, respectively. They also demonstrated that the WSS of a tapered artery (maximum 150 Pa) is higher than that of a straight artery model (maximum 122 Pa). Similar observations are obtained in our study. In our models, the stenosis levels for the lipid-only and calcification-only models are $61\%$ and $43\%$, respectively. The maximum WSS at the peak systole of the lipid-only model is higher than that of the calcification-only model (Fig. 6). Moreover, the studied arterial wall is in a tapered shape in general (diameters of the inlet and outlet artery are about 9.01 mm and 6.93 mm), which also contribute to the high WSS in our models.

Figure 7 shows the time evolutions of blood pressure on the stenosis surfaces (p1 and p2). Due to the flow domain reduction in calcification-lipid model, the pressures on both points are lower in the model with two plaques (calcification-lipid model) than that in the model with one plaque (calcification-only model and lipid-only model).

von Mises stress (VMS) is widely accepted as a useful measurement indicates the vulnerability of atherosclerotic plaques (Humphrey and Holzapfel 2012; Creane et al. 2010; Gao et al. 2009). The VMS distributions at peak systole show that the plaque stress is lower than the stress of the artery wall (Fig. 8). This observation coincides with previous studies (Tang et al. 2004, 2005), which state that plaques reduce the lumen area in the stenosis region and leads to lower stress in the plaque. Additionally, Fig. 8b, d, f and g illustrates that plaque stress concentrates near the plaque shoulder, which is commonly considered as the plaque rupture location (Falk et al. 1995). This result is also in good agreement with observations in literatures (Creane et al. 2010; Li et al. 2006).

The time evolutions of MVMS (Fig. 9) demonstrate that the stresses of calcified and lipid plaques diverge notably, and the maximum stress of lipid plaque (118.7 kPa) is higher than the maximum stress of calcified plaque (65.3 kPa). This result is in good agreement with the result in the literature (Karimi et al. 2014a), which reveal that the VMS of calcified plaque is the lowest among all plaque types. In terms of the soft nature of lipid-rich plaque with the higher stress acted on it, the results from this study indicated that lipid plaque has a higher risk to rupture. Moreover, the MVMS of calcified and lipid plaques in the model with two plaques (calcification-lipid model in Fig. 9) is lower than the MVMS of the corresponding plaques in the models with just one plaque (calcification-only model and lipid-only model in Fig. 9), which is coincident with the measurement of flow pressure on the stenosis surface (Fig. 7). The presence of two plaques diminishes the flow domain and decreases the loading force on the plaque (calcified or lipid), which leads to a reduction in the plaque (calcified or lipid) stress.

A limitation of this model is the isotropic material model used for the femoral artery. The arterial wall is consist of smooth muscle cells, elastin and collagen fibrils. Hence, a fibre-reinforced composites model, which assuming collagen fibres are embedded in a matrix, provides a constitutive model describing more realistic biomechanical properties of an arterial wall. However, the Mooney–Rivlin material model is widely used in finite element simulations for stenosed arteries (Kim et al. 2008; Tang et al. 2017; Wang TDMAea L, 2019). In this study, Mooney–Rivlin material property based on tensile tests of human femoral blood vessels is employed for the arterial wall from the previous study (Prendergast et al. 2003), who showed that this material model is adequate to capture the elastic behaviour of the tested tissue. As the purpose of this study is to compare the effect of multiple plaques in patient-specific arterial models, the Mooney–Rivlin material property employed for the artery is acceptable.

## Conclusion

In conclusion, we perform FSI simulations for human stenosed femoral artery in three models to compare the biomechanical features of femoral artery models with different types of plaques. The WSS around the calcified plaque (low stenosis degree) is lower than that around the lipid plaque (high stenosis degree). Therefore, new plaque might be further accumulated around the calcified plaque compared with the lipid plaque. Conversely, the lipid plaque causes oscillatory WSS in the healthy region behind it, which indicate that the initiation of new plaques may happen in the healthy area behind the lipid plaque. In addition, structure analyses indicate that maximal stress principally sits in the plaque shoulder, where it is commonly accepted as the plaque rupture location. Lastly, the lipid plaque, accompanied by the calcified plaque around, might reduce its risk of rupture due to the reduced the stress acting on it.

## Data Availability

All data generated or analysed during this study are included in this published article.
